# Main factors affecting the efficacy of medicinal plants during the cultivation process

**DOI:** 10.3389/fpls.2025.1634926

**Published:** 2025-09-16

**Authors:** Chunhong Zhang, Xuejing Zhong, Saqirula Bao, Myadagbadam Urtnasan, Minhui Li

**Affiliations:** ^1^ College of Pharmacy, Baotou Medical College, Baotou, China; ^2^ Inner Mongolia Key Laboratory of Characteristic Geoherbs Resources Protection and Utilization, Baotou Medical College, Baotou, China; ^3^ Inner Mongolia Institute of Traditional Chinese Medicine, Hohhot, China; ^4^ Institute of Traditional Medicine and Technology of Mongolia, Ulaanbaatar, Mongolia

**Keywords:** medicinal plants, ecological modulators, bioactive compounds, cultivation standardization, quality assurance

## Abstract

The escalating global demand for safe and efficacious traditional Chinese medicine (TCM) has underscored the urgent need for a stable and high-quality supply of medicinal plant resources. While large-scale cultivation offers a practical solution to alleviate the pressure on wild populations, ensuring the consistent expression of active secondary metabolites and reproducible pharmacological efficacy remains a central challenge. The biosynthesis and accumulation of these bioactive compounds are governed by a complex interplay of factors spanning all stages of cultivation and post-harvest handling. This narrative review systematically analyzes and identifies from current literature the five key dimensions influencing the therapeutic efficacy of medicinal plants: (1) production area selection, where ecological suitability shapes plant metabolic responses; (2) genetic resources, which determine the biosynthetic potential of active constituents; (3) field management practices, including nutrient regulation and cropping systems; (4) the control methods of diseases, insect pests, and weeds, integrating ecological approaches with rational chemical use to reduce residues and ensure safety; and (5) harvesting, processing, and storage techniques, which affect the stability and bioavailability of bioactive compounds. These interconnected factors collectively determine the final quality and therapeutic efficacy of medicinal plant raw materials. Coordinated management of these five dimensions is essential for establishing standardized, ecologically sustainable cultivation systems, which are crucial for ensuring quality assurance, enhancing supply chain resilience, and promoting the modernization and global recognition of TCM. This review offers a comprehensive theoretical and technical framework to guide the development of efficient medicinal plant production systems yielding materials with consistent efficacy and safety.

## Graphical Abstract

The medicinal plant production optimization framework integrates five sequentially linked components: production area selection, genetic resources, field management or cultivation management, the control method of disease, insect pest and weeds, and harvesting processing storage, with their synergistic interaction enhancing medicinal material quality consistency and therapeutic potency. MMS, Multi-Marker Scanning; GLM, Generalized Linear Model; QTL, Quantitative Trait Locus; PhyDA, Phylogenetic Diversity Analysis.

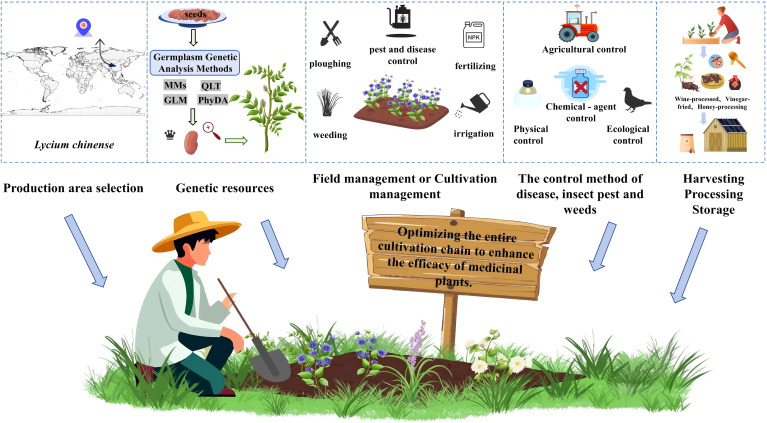

## 1 Introduction

The earliest written records of medicinal plants can be traced back to the *Ebers Papyrus* from ancient Egypt, dated around 1600 BCE, and *Ayurveda* from India, both of which have a history of over 3,000 years. According to statistics, there are approximately 374,000 known plant species worldwide, among which 28,000 are classified as medicinal plants (Fazan et al., 2020; Zhang et al., 2025); medicinal plants hold an irreplaceable position in traditional medical systems worldwide, with 80% of the global population relying on them for disease prevention and treatment (Thokchom et al., 2023). Currently, approximately 900 species of medicinal plants are widely traded as commodities worldwide (Van Wyk and Wink, 2017).

With the increasing demand for medicinal plants, artificial cultivation has become a primary solution to address the growing shortage of wild medicinal plant resources. The proportion of cultivated plant-based medicinal materials in the market has grown rapidly, making it a key focus for researchers to ensure the efficacy of cultivated medicinal plants. Numerous studies have shown that factors such as cultivation region, genetic resources, cultivation techniques, and post-harvest processing and storage can significantly influence medicinal efficacy. The objective of this review is to systematically summarise and analyse the key factors influencing the content of bioactive compounds in medicinal plants. Synthesising extant research findings, it aims to provide a scientific basis for quality control and efficacy assurance of artificially cultivated medicinal plants, thereby promoting the sustainable utilisation of medicinal plant resources and the healthy development of the industry.

## 2 Production area selection

The quality and yield of medicinal plants are significantly influenced by environmental factors, which regulate their growth, development, and metabolic processes. Research has demonstrated that moderate environmental stresses, including variations in temperature, light intensity, and water availability, can stimulate the production of secondary metabolites. These compounds represent the active pharmaceutical ingredients that are responsible for the therapeutic effects of medicinal plants. In contrast, unfavourable environmental factors have been shown to impede the accumulation of biomass and to reduce the concentration of active constituents. This, in turn, has been demonstrated to diminish the quality and efficacy of medicinal materials. According to statistics, more than 214,000 secondary metabolites have been identified (Pant et al., 2021). Medicinal plants from different regions experience variations in environmental factors such as climate, topography, and soil. Even for the same species, different environmental stresses can induce the formation of distinct secondary metabolites. Specific environmental stresses promote the accumulation of particular secondary metabolites, providing valuable guidance for selecting cultivation sites for medicinal plants based on their desired efficacy (Pant et al., 2021). The chemical composition and pharmacological potential of the leaves and pseudobulbs of *Prosthechea karwinskii*, a species of *Orchidaceae* endemic to Mexico, show significant geographical differences among different production areas in Oaxaca State, Mexico (Barragán-Zarate et al., 2025). The key bioactive components and pharmacological effects of *Pimpinella anisum* L. from Iran and Portugal also differ significantly (Farzaneh, 2016). Both studies demonstrate that environmental factors associated with production areas are decisive in influencing the active constituents of medicinal plants. In practical production, it is highly recommended that cultivation be guided through the study of medicinal plant suitability zoning. Selecting plots with suitable ecological factors, medicinal components that meet therapeutic requirements, and compatibility with cultivation and production purposes is one of the key factors for success (Zhang M. et al., 2021; Shang et al., 2023; Rong et al., 2024). Furthermore, when selecting production areas, a pollution avoidance mechanism must be established by prioritizing regions remote from urban industrial zones, major transportation arteries, and known pollution sources (e.g., chemical manufacturing plants; municipal landfills) (Twumasi et al., 2021). Subsequently, the processing radius should be incorporated as a key metric: reducing the distance between cultivation sites and processing or drying facilities decreases transportation losses and ensures raw material freshness and production efficiency (Tegtmeier et al., 2023; Wang et al., 2023). Finally, evaluations must concurrently address the impact of cultivation zones on phytomedicine safety. For example, pesticide residues and heavy metal contamination are major factors affecting the quality of *Lycii Fructus* (*Lycium barbarum* L.). However, products cultivated in the Qaidam Basin of Qinghai Province, China, have largely avoided this issue due to appropriate site selection. This case can be promoted as a successful example of cultivation site selection. Qinghai Province is not a traditional producing area for *Lycii Fructus*. Owing to its exceptionally clean and unpolluted natural environment, the region is regarded as one of the world’s four ultra-clean zones. Furthermore, its high elevation and low temperatures contribute to the minimal incidence of pests and diseases. As a result, no pesticides or chemical fertilizers are used in the cultivation process, and the products are free from heavy metals or pesticide residues. The *Lycii Fructus* produced in this region are of exceptional quality, and their green, organic, and pollution-free attributes have earned them high recognition in the industry (Luo, 2019).

## 3 Genetic resources

Genetic resources are the foundation of medicinal plant cultivation and production, serving as the internal factor affecting the plants’ medicinal efficacy. High-quality germplasm resources are the primary condition and important evaluation criterion for ensuring medicinal efficacy. First, identifying the source of medicinal materials and determining the correct species is crucial. Different plant species tend to vary significantly in the types and quantities of secondary metabolites they synthesize and accumulate, attributable to their inherent genetic characteristics. This variation remains pronounced even among closely related species used for the same medicinal purpose. For instance, *Bupleurum scorzonerifolium* Willd. and *Bupleurum chinense* Franch., both serving as the botanical sources for the traditional medicinal herb *Bupleuri Radix*, demonstrate a 4- to 5-fold difference in the combined content of Saikosaponins A, C, and D between these two species (Chen, 2024).

Within a single species, genetic diversity is evident in variations in genetic composition among populations and among individuals within them. This diversity is fundamental basis for biological adaptation and evolution, and is driven by environmental factors or human interventions (e.g., cultivar selection). These genetic variations directly alter phenotypic traits in medicinal plants while significantly modulating the content of bioactive compounds. Numerous studies have confirmed a significant correlation between the phenotypic characteristics of medicinal plants and the accumulation of bioactive compounds (Wang et al., 2019; Yuan et al., 2023; Shi et al., 2024). Analysing this relationship allows accurate and rapid prediction of the internal quality of medicinal materials based on the external traits of source plants. It is expected that applying phenotype-assisted selection strategies will accelerate the breeding of superior medicinal plant varieties. At present, research in this field is still in its early exploratory stage, with limited systematic studies. However, progress has already been made in several medicinal plants such as *Panax ginseng* C. A. Mey. and *Panax notoginseng* (Burkill) F. H. Chen ex C. H. Chow, and it is expected to become a focal point of future research. Currently, most medicinal plant cultivation still relies on wild types or local varieties that have not been selectively bred. The development of new cultivars is still in its early stages. According to statistics, by 2020, only 225 new cultivars of medicinal plants had been registered; of which only a few had reached large-scale production (Wang et al., 2020). The future trend is to cultivate superior varieties with medicinal components that meet the requirements, strong resistance, and high yields, through the integration of modern biotechnology and traditional breeding techniques. However, it is important to emphasize the evaluation work before new varieties are introduced into production. While focusing on medicinal efficacy, issues such as ecological, environmental and genetic safety be given due consideration. For instance, techniques like mutagenesis and transgenic breeding can induce genetic alterations in species (Stadler, 1928; Paterniani, 2006; Marone et al., 2023). This prompts critical questions regarding the creation of new varieties of medicinal plants or entirely new species. Furthermore, it is crucial to ascertain whether such genetically modified organisms pose a threat to the environment or other species. These issues demand prioritised research focus and practical resolution in the future.

## 4 Field management or cultivation management

Field management involves the cultivation of medicinal plants during their growing period of medicinal plants, including tasks such as ploughing, weeding, fertilising, irrigating and controlling pests and diseases. These cultivation practices, whether applied individually or in combination, influence the biosynthesis and accumulation of secondary metabolites in medicinal plants, affecting their therapeutic efficacy. For instance, the regulation of cultivation parameters, including temperature, light, humidity, and soil fertility, can significantly influence the concentration and profile of chamomile essential oil, the bioactive compounds derived from *Matricaria chamomilla* L (Subhdara, Jakhmola and Panthari, 2024). Compared to plastic film-covered cultivation, open-field cultivation of *Thymus vulgaris* L. leads to a higher accumulation of phenolic acids (Kosakowska et al., 2021). In practical cultivation management, it is essential to avoid continuous cropping. Continuous cropping can alter the physicochemical properties of the soil, reduce the abundance of beneficial microorganisms, and release allelopathic compounds. These changes, in turn, negatively impact the growth and development of medicinal plants, as well as the synthesis and accumulation of bioactive compounds. Even with appropriate cultivation measures, the plants may still exhibit reduced vigour, with an increased frequency of pest and disease occurrences, and the yield and quality of the medicinal materials may not be guaranteed (Zhuang and Chen, 2015; He et al., 2021; You et al., 2024).

Furthermore, nutrient management plays a crucial role in the quality of medicinal plants. Proper fertilization typically enhances the content of bioactive compounds in medicinal plants, but excessive or imbalanced fertilization can have the opposite effect. In practical production, soil testing and formula-based fertilization are recommended (Król et al., 2020; Paschalidis et al., 2021; Hao et al., 2024); for example, research by Skubij et al. indicates that the amount and type of potassium fertilizer significantly affect the content of the active ingredient L-ascorbic acid in *Ocimum basilicum* L (Dzida et al., 2018). High nitrogen fertilizer application reduces the content of total phenols, total flavonoids, and cynandione A in the aerial parts and tubers of *Cynanchum boudieri* H. Lév. & Vaniot. In contrast, the content of cynandione A increases with higher potassium fertilizer application rates (Tseng et al., 2022). Currently, organic fertilizers, green manure, and microbial inoculants are gradually replacing traditional chemical fertilizers. These alternatives can improve the physicochemical properties of the soil, modify root exudation patterns, and alter microbial communities, thereby enhancing the biomass and quality of medicinal plants. At the same time, they can also mitigate the adverse effects of continuous cropping (Darakeh et al., 2021; Liang et al., 2021; Ram et al., 2024).

## 5 The control methods of disease, insect pests and weeds

In the cultivation of medicinal plants, pesticides are often used to prevent, eliminate, or control diseases, insect pests and weeds, ensuring and promoting the growth and development of the plants. However, improper pesticide application can negatively impact the quality of medicinal plants. For example, empirical evidence confirms that non-standard pesticide application during *P. notoginseng* cultivation causes a 13.82% reduction in notoginsenoside content (Kang et al., 2025). At the same time, there is a risk of pesticide residues exceeding safety standards. Many countries have established lists of prohibited pesticides for medicinal plant cultivation. However, the availability of targeted, highly efficient, and environmentally friendly pesticides remains limited. The currently favoured approach to pests, disease, and weeds control emphasizes prevention as the primary strategy, with agricultural, ecological, and physical control methods given priority and chemical pesticide control as a supplementary measure. Additionally, it is advisable to use pesticides that are highly specific, efficient, low in toxicity, and characterized by short environmental half-lives. Some studies further suggest that the presence of moderate weeds populations may have minimal negative effects on medicinal plant yield while potentially reducing pests and disease pressure, enhancing the quality of medicinal materials, and lowering input costs. This cultivation strategy represents a promising direction for sustainable production and merits further in-depth investigation.

## 6 Harvesting, processing, and storage

The chemical composition of medicinal plants undergoes dynamic changes during growth (Hazrati et al., 2024). For example, harvesting *Fritillaria cirrhosa* D. Don at the late flowering stage results in the highest total alkaloid content (Ma et al., 2021). The highest essential oil content for the four medicinal plants, dill (*Anethum graveolens* L.), parsley (*Petroselinum crispum* (Mill.) Fuss), coriander (*Coriandrum sativum* L.), and mint (*Mentha × piperita* L.), is obtained when harvested at 9 weeks, 19 weeks, 3 weeks, and 6 weeks after sowing, respectively (El-Zaeddi et al., 2020). After harvesting, prompt and appropriate processing is critical to preserving the quality of medicinal materials. Standard post-harvest procedures typically include cleaning, trimming, and drying. For certain species, specialized treatments—such as steaming, boiling, blanching, or “sweating”—are necessary prior to drying due to their unique characteristics (World Health Organization, 2003; Xu et al., 2020; Dehel Gamage et al., 2021; Kirkwood et al., 2023; Ghorbani et al., 2025; Sasaki et al., 2025). Notably, variations in processing methods can significantly influence the therapeutic efficacy of medicinal plants (Qiao et al., 2024). For example, Sałata et al. (2020) compared the drying of *Lavandula angustifolia* Mill. under natural conditions and at 30°C. The results showed that the drying at 30°C increased the average essential oil content by 18%, total phenolic acids by 50%, and exhibited higher antioxidant activity. Similarly, the storage process is also an important factor that affects the efficacy of medicinal plants. Inappropriate environmental conditions and prolonged storage times can cause the gradual degradation of bioactive compounds, resulting in a loss of therapeutic effects. Moreover, high temperatures and high humidity conditions can promote the growth of harmful substances, such as aflatoxins, ochratoxins, and zearalenone, which are fungal toxins produced by fungi such as *Aspergillus* and *Fusarium* (Zhang T. et al., 2021). For example, Ndoro et al. conducted a study on fungal toxins in eight commonly traded South African medicinal plants and found widespread contamination that is likely to pose a threat to consumer health (Ndoro et al., 2022). Additionally, the analysis by Lee et al. of 729 samples from 19 medicinal plants in South Korea revealed the presence of aflatoxins in 124 samples (Lee et al., 2014). Therefore, it is imperative to establish a systematic and comprehensive monitoring mechanism for mycotoxins, enhance human exposure assessment based on biomarkers, and accelerate the improvement of relevant laws and regulatory standards.

In conclusion, every step of the cultivation process of medicinal plants, from selecting the cultivation site, choosing plant material, planting, harvesting, processing, to storage, can affect their therapeutic efficacy. A practical approach to production involves developing personalized cultivation plans based on prior research and actual production conditions, strengthening monitoring and management, conducting strict testing, protecting the ecological environment, and adhering to ethical standards. Ultimately, this ensures the production of products that meet therapeutic requirements. Building on the WHO’s list of commonly used medicinal plants (World Health Organization, 2006), this paper provides a comprehensive analysis and summary of how cultivation and processing approaches enhance the therapeutic efficacy of selected medicinal plant species (**Supplementary Table S1**). The study aims to establish a scientific basis for standardized cultivation and quality control practices, with all investigated herbal materials having their botanical origins validated against this authoritative list.
