# Supplementary material for: Main factors affecting the efficacy of medicinal plants during the cultivation process
Source: Front Plant Sci. 2025 Sep 16;16:1634926. doi: 10.3389/fpls.2025.1634926 (PMC12479407; doi:10.3389/fpls.2025.1634926)
Supplement: Supplementary file 1 [file Table1.docx]

Supplementary Material

**Supplementary Table 1.** Cultivation and Processing Approaches for Improving the Therapeutic Efficacy of Medicinal Plants

| Medicinal material names | Plant part | Original plant | Cultivation practices | Processing methods | Reference |
| --- | --- | --- | --- | --- | --- |
| Bulbus Allii Cepae | Fresh or dried bulbs | *Allium cepa* L. | Selecting red-skinned cultivars with high dry matter content promotes the accumulation of saccharides and flavonoids, while white-skinned dehydrator-type cultivars favor the synthesis of S-substituted cysteine conjugates (ACSOs). Planting schedules should be optimized according to climatic conditions. Although cultivation methods have minimal impact on efficacy, local adaptation is recommended. Application of low-dose sulfur-bentonite fertilizer enriched with orange residue significantly enhances the content of bioactive compounds such as chlorogenic acid. | - | [1,2] |
| Bulbus Allii Sativi | Fresh or dried bulbs | *Allium sativum* L. | Avoiding nitrogen fertilization and applying moderate levels of sulfur—particularly a high-sulfur and low-nitrogen combination during the main growth to early maturity stages—can significantly enhance alliin content in garlic bulbs; meanwhile, moderate drought stress is beneficial for promoting fructan accumulation. | - | [3,4] |
| Aloe | Juice of leaves (dried) | *Aloe vera* (L.) Burm. f. | Transplanting should be avoided in winter and can be carried out during the rest of the year. Surface tillage is recommended to prevent soil erosion, and good drainage throughout the soil profile must be ensured to avoid waterlogging and disease. Partial shading during the seedling stage can increase leaf number. A row spacing of 90–100 cm and plant spacing of 45–50 cm are recommended, with a planting density of 20,000–25,000 plants/ha. Manual weeding is preferred, and regular rainfall or irrigation is required. | Post-harvest processing includes raw material reception, unloading, weighing, storage, initial washing, secondary washing, removal of leaf tips and bases, leaf filleting, and removal of the outer leaf skin. | [5] |
|  |  | *Aloe ferox* Mill. |  |  |  |
| Aloe Vera Gel | Leaf gel | *Aloe vera* (L.) Burm. f. |  |  |  |
| Radix Astragali | Dried root | *Astragalus membranaceus* (Fisch.) Bunge | The content of Astragalus polysaccharides is mainly influenced by temperature and precipitation, while the accumulation of astragaloside IV is affected by multiple factors including soil properties, solar radiation, altitude, temperature, and precipitation. Ecological environment regulation is key to improving active compound content. Saponins accumulate predominantly in the early growth phase, whereas flavonoids accumulate later, suggesting that staged harvesting could be considered. | Fresh-cut Astragali Radix slices generally contain higher levels of calycosin-7-O-glucoside than traditional dried-cut ones. Currently, processing is shifting toward integration at the production site, where fresh roots are sliced and dried directly, reducing repeated moistening and drying, and minimizing active ingredient loss. Honey-processing remains the main method for auxiliary treatment. | [6-[7][8][9]10] |
|  |  | *Astragalus membranaceus* var. *mongholicus* (Bunge) P.K.Hsiao |  |  |  |
| Fructus Bruceae | Dried ripe fruit | *Brucea javanica* (L.) Merr. | The roots of the plant can be harvested throughout the year, while the fruits are mainly harvested in autumn upon maturation. Fruits of preferable quality are large, intact, and plump, with white kernels and high oil content. After harvesting, impurities are removed and the fruits are dried. | Traditionally, processing involves shelling to obtain the kernels, followed by frosting and pounding to remove oil. In modern practices, more emphasis is placed on removing fruit shells and impurities to ensure product purity. | [11] |
| Radix Bupleuri | Dried root | *Bupleurum falcatum* L. | The total content of saikosaponins in *Bupleurum* shows a significant positive correlation with longitude, latitude, and altitude. The monthly average temperature and surface temperature are the main climatic factors affecting saikosaponin accumulation, followed by sunshine duration. Low temperature, drought, and longer sunlight exposure are conducive to the enrichment of saikosaponin components. Cultivation temperature should be controlled within a moderate low-temperature range of 16.5–17.6°C, and high temperatures (such as 21.0°C) should be avoided. Regarding soil properties, high-saikosaponin mainly grows in soils with low alkalinity and low nitrogen content. High nitrogen levels significantly increase the yield and quality of the aerial parts (especially flowers), but have limited effects on the roots; nitrogen fertilizer can promote the accumulation of saikosaponin A while inhibiting the content of saikosaponins C and D. | Vinegar-processed Bupleurum Radix is the most widely used processed form of Bupleurum Radix in clinical practice, and air-drying is considered the most suitable method for Bupleurum raw materials. The optimal origin processing technique involves cleaning the Bupleurum, steaming it for 30 minutes, slicing it into 2–4 mm thick pieces, and then drying them. To reduce costs, it is more reasonable and appropriate to cut fresh materials into a semi-dried state with about 50% moisture content before air-drying. | [12-[13][14][15]16] |
|  |  | *Bupleurum scorzonerifolium* Willd. |  |  |  |
| Herba Centellae | Dried aerial parts or whole plant | *Centella asiatica* (L.) Urban | The annual temperature range, annual precipitation, precipitation of the wettest month, precipitation seasonality, and precipitation of the coldest quarter at the origin significantly affect the content of active compounds. The optimal light condition is a light intensity of 200 µmol·m⁻²·s⁻¹ with a red to blue light ratio of 8:2. | The suitable drying method for Centella at the production site is hot air drying at 70°C. | [17-[18]19] |
| Flos Chamomillae | Dried flowering heads | *Chamomilla recutita* (L.) Rauschert （syn. *Matricaria chamomilla* L.） | Before sowing or transplanting, the field should be ploughed 2–3 times with a mouldboard plough to ensure the soil is loose, friable, and level, and to completely remove weeds and residues of the previous crop. If soil moisture is insufficient, pre-sowing irrigation should be applied. During the final ploughing, well-decomposed organic manure—such as farmyard manure, poultry manure, or vermicompost—should be incorporated into the soil to improve its structure and nutrient supply. In the case of seedling transplantation, it is recommended to apply basal manure 30 days prior to transplanting. For details on planting density, intercropping systems, nutrient and water management, weed control, pest and disease management, please refer to the relevant literature. | Post-harvest processing mainly involves removing impurities from the harvested flower heads, followed by shade drying for 3 to 4 days. While sun drying can promote the formation of chamazulene, strict control of the drying environment throughout the entire process is essential to ensure product quality and the stability of active compounds. | [20] |
| Cortex Cinnamomi | Inner bark of shoots | *Cinnamomum verum* J.S. Presl. | The optimal harvesting time for *Cinnamomum cassia* is 120 months (i.e., 10 years) after planting. The accumulation of its medicinal components is also significantly influenced by factors such as germplasm resources, genetic background, mean annual rainfall, and temperature. | - | [21,22] |
|  | Cork-free trunk bark | *Cinnamomum burmanni* (Nees & T.Nees) Blume (syn. *Cinnamomum cassia* Nees ex Blume) |  |  |  |
| Rhizoma Coptidis | Rhizoma | *Coptis chinensis* Franch. | The rhizome length, diameter, and weight of *Coptis chinensis* all increase with the years of growth, with the rhizome length reaching its maximum at 4 years old, while the rhizome diameter and weight peak at 5 years old. | In ancient times, sun-drying and roasting were the main methods used for origin processing. In modern practice, techniques such as shaded drying under thin paper, hot-air drying, oven drying, and low-heat stir-drying have been adopted. As direct sunlight and roasting significantly affect the content of berberine, strict temperature control is required during the drying process to ensure the stability of active compounds and the quality of the medicinal material. | [23,24] |
|  |  | *Coptis deltoidea* C. Y. Cheng & P. G. Xiao |  |  |  |
|  |  | *Coptis japonica* (Thunb.) Makino (syn. *Coptis japonica* Makino) |  |  |  |
| Rhizoma Curcumae Longae | Dried rhizome | *Curcuma longa* L. | The optimal harvest time for turmeric rhizome yield is between 5 to 9 months after planting, and the most suitable soil pH is approximately 5.2. | The sliced shade-drying method preserves the highest content of curcuminoids, with superior color and aroma, and is therefore recommended as the optimal processing method at the production site. | [25-[26][27]28] |
| Radix Echinaceae | Fresh or dried roots | *Echinacea angustifolia* D.C. var. *angustifolia* | Plants of the genus *Echinacea* prefer full sunlight, are tolerant to heat and drought, and have strong adaptability; they are best cultivated in well-ventilated, sunlit, and fertile sandy loam soils, and can be propagated by seeds, seedlings, or root cuttings. During the early growth stage, timely weeding and fertilization are needed. The aerial parts are generally harvested after two years, while the underground parts require more than three years of growth before harvest. | - | [29] |
|  |  | *Echinacea angustifolia* var. *strigosa* McGregor |  |  |  |
|  |  | *Echinacea pallida* (Nutt.) Nutt. |  |  |  |
| Herba Echinaceae Purpureae | Fresh or dried aerial parts (in full bloom) | *Echinacea purpurea* (L.) Moench | Similar to the cultivation conditions of Radix Echinaceae, *Echinacea purpurea* (L.) Moench shows stronger tolerance to drought and waterlogging, and short-term water stress before medicinal harvest can enhance the chicoric acid content in its leaves. | - | [30] |
| Herba Ephedrae | Dried stem or aerial part | *Ephedra sinica* Stapf | The total alkaloid content and composition ratio of *Ephedra sinica* tend to stabilize from the second year onward, with dry weight remaining stable from the fourth year. Moreover, the composition ratio of ephedrine alkaloids is primarily controlled by genetic factors and is minimally affected by environmental conditions or changes in the growth cycle. | Honey-frying is the main processing method for Ephedra sinica, with specific techniques varying across different periods (see reference 33). | [31-[32]33] |
| Folium Ginkgo | Dried whole leaf | *Ginkgo biloba* L. | *Ginkgo biloba* is suitable for growing in fertile, well-aerated soils with moderate nutrient levels, and during cultivation, pest and disease control is rarely needed; different treatments of nitrogen, phosphorus, and potassium fertilizers have significant effects on the nutrient content of Ginkgo leaves: applying phosphorus fertilizer alone can increase the phosphorus content in the leaves, while applying potassium fertilizer alone can significantly increase the nitrogen and potassium contents in the leaves. | *-* | [34,35] |
| Radix Ginseng | Dried root | *Panax ginseng* C.A. Meyer | *Panax ginseng* is a typical semi-shade perennial plant, and its growth is greatly influenced by light intensity. The optimal temperature range for its growth is 20–25 °C, and the optimal soil moisture range is 60%–80%. | Steaming ginseng at a high temperature (120 °C for 2 hours) is the optimal processing method, as it significantly enhances its antioxidant activity and vasorelaxant effects, and leads to the formation of high levels of rare ginsenosides Rg3 and Rg5. | [36,37] |
| Radix Glycyrrhizae | Dried roots and rhizomes | *Glycyrrhiza glabra* L. | *Glycyrrhiza spp.* grows best in deep, well-drained sandy loam soils with an annual mean temperature of 5–25 °C, annual rainfall ranging from 400 to 1160 mm, and a soil pH of 5.7–8.2. It is drought-tolerant and light-loving. Spring is the optimal season for sowing, and a planting density of 24,000–42,000 plants per hectare is recommended. Proper irrigation and low-dose nitrogen and phosphorus fertilization can effectively promote root development. In addition, symbiosis with mycorrhizal fungi can significantly enhance yield and the accumulation of active compounds such as glycyrrhizin. | The common processing method for licorice is honey-frying. Refined honey is diluted with boiling water and mixed with licorice slices, then infused in a closed container and gently fried over low heat until yellow or deep yellow, followed by cooling. Sulfur fumigation should be avoided. | [38-[39]40] |
|  |  | *Glycyrrhiza uralensis* Fisch. |  |  |  |
| Radix Paeoniae | Dried root | *Paeonia lactiflora* Pall. | Selection of a growing environment with adequate annual precipitation, relatively low average temperature, and rich in zinc and potassium is recommended. Roots should be harvested at noon during the sprouting or flowering stage in spring, after 4–5 years of cultivation. Leaves are best collected during the early stages of plant development. | For post-harvest processing, freeze-drying is preferred over high-temperature drying. | [41-[42]43] |
| Semen Plantaginis | Dried, ripe seed | *Plantago afra* L. | Use aeroponic cultivation to ensure adequate water and nutrient supply and good root aeration. Under aeroponic conditions, roots should be harvested at noon during the budding or flowering stage in spring, approximately 2 months after sowing; under soil cultivation, 4–5 months of growth is required. Leaves should be collected during the vegetative growth stage. Moderate environmental stress is conducive to the accumulation of active compounds. A slightly suboptimal ecological environment with mild stress is recommended to balance biomass production and secondary metabolite content. | Post-harvest processing should avoid high-temperature drying; freeze-drying is preferred to preserve nutritional components. | [44,45] |
|  |  | *Plantago indica* L. |  |  |  |
|  |  | *Plantago ovata* Forsk. |  |  |  |
|  |  | *Plantago asiatica* L. |  |  |  |
| Radix Platycodi | Root | *Platycodon grandiflorus* (Jacq.) A. DC. (syn. *Platycodon grandiflorum* (Jacq.) A. DC.) | Prefer a 3-year growth period with harvesting in September to October. Select suitable production regions to improve medicinal quality. | Dry at 80 °C to preserve active compound content. | [46,47] |
| Radix Rauwolfiae | Dried root | *Rauvolfia serpentina* (L.) Benth. ex Kurz | The optimal harvest time is in winter (December) after four years of growth following transplantation, when fresh root weight and the contents of rhynchophylline and isorhynchophylline reach their peak, resulting in the best overall yield and active ingredient performance. Regarding fertilization, moderate application of nitrogen, phosphorus, and potassium (N2P2K2 treatment) significantly promotes plant height, fresh root weight, and the accumulation of rhynchophylline and isorhynchophylline, achieving maximum effects. | Washing in a drum mixer for 30 minutes is an efficient and feasible root processing method, and the thickness of the root segments does not affect the quality of the crude drug. | [48,49] |
| Rhizoma Rhei | Underground parts (rhizome and root) | *Rheum officinale* Baill. | The recommended nitrogen fertilizer application rate was N₂ level (150 kg·ha⁻¹). Meanwhile, moderate drought stress can promote the accumulation of active compounds in plants through physiological induction and enhanced interaction with beneficial microbes. | Rhubarb should be dried at a moderate temperature, with studies indicating that the drying temperature of *Rheum palmatum* and *Rheum officinale* should not exceed 50°C. Moistening treatment following fresh slicing and drying can enhance anthraquinone content but may lead to a decrease in phenolic acid levels. | [50-[51]52] |
|  |  | *Rheum palmatum* L. |  |  |  |
| Folium Sennae | Dried leaflets | *Cassia senna* L. | The application of two novel fertilizers—namely, enriched compost produced by composting the distillation waste of aromatic plant biomass through a natural process and blending it with mineral powder, and enriched biochar-mineral material prepared by blending biochar (derived from the same distillation waste via hydrothermal reaction) with mineral powder—significantly increases the content of bioactive compounds in senna pods compared to chemical fertilizer treatment. | Regular monitoring of aflatoxin levels in senna pods from harvest to storage is a key measure to ensure their quality and food safety. | [53,54] |
| Fructus Sennae | Dried ripe fruit |  |  |  |  |
| Herba Thymi | Dried leaves and flowering tops | *Thymus vulgaris* L. | Optimal cultivation requires well-drained calcareous soil under full sun exposure, avoiding saline conditions. Planting should occur in temperate or warm-temperate regions with sowing conducted from late winter to early spring. Apply an annual balanced basal fertilizer (N-P-K-S), supplementing with nitrogen after each harvest to promote new shoot growth while avoiding excess. Harvesting should target the full bloom to early fruiting stages. | After sorting and selecting, Herba Thymi is first treated with 1-MCP, then washed in a sodium hypochlorite solution (pH 10.33, ORP 650 mV, ACC 100 mL/L) for 10 minutes, followed by rinsing with water and centrifugation for dehydration. Finally, it is packaged in polyethylene bags with a thickness of 32 μm and stored at 4°C. | [55,56] |
|  |  | *Thymus zygis* L. |  |  |  |
| Radix Valerianae | Dried rhizomes, roots and stolons | *Valeriana officinalis*L. | The establishment of plantations through direct seeding is fully justified. Optimal sowing should target mid-August, though alternative sowing or planting dates may be utilized under favorable weather conditions; the critical factor for success remains adequate humidity during germination and initial plant development. | - | [57] |
| Rhizoma Zingiberis | Dried rhizome | *Zingiber officinale* Roscoe | *Zingiber officinale* achieves peak essential oil accumulation at the 5-month harvest stage, beyond which yield significantly declines with increasing maturity. | *I*ntermittent microwave combined with convective drying (IM&CD), due to its high efficiency, good quality retention, and lower cost, is the optimal drying method for ginger rhizome and other thermo-sensitive products. | [58,59] |

**References**

1. Böttcher, C., Krähmer, A., Stürtz, M., Widder, S., and Schulz, H. (2018). Effect of Cultivar and Cultivation Year on the Metabolite Profile of Onion Bulbs ( *Allium cepa* L.). *Journal of agricultural and food chemistry*, 66(12), 3229–3238. https://doi.org/10.1021/acs.jafc.8b00254
2. Matrella, M. L., Valletti, A., Marra, F., Mallamaci, C., Cocco, T., and Muscolo, A. (2022). Phytochemicals from Red Onion, Grown with Eco-Sustainable Fertilizers, Protect Mammalian Cells from Oxidative Stress, Increasing Their Viability. *Molecules*, 27(19), 6365. https://doi.org/10.3390/molecules27196365
3. Zhou, Q., Sun, H., Zhang, G., Wang, J., and Tian, J. (2023). Gene Co-Expression Analysis Reveals the Transcriptome Changes and Hub Genes of Fructan Metabolism in Garlic under Drought Stress. Plants, 12(19), 3357. https://doi.org/10.3390/plants12193357
4. Bloem, E., Haneklaus, S., and Schnug, E. (2010). Influence of fertilizer practices on S-containing metabolites in garlic (*Allium sativum* L.) under field conditions. *Journal of agricultural and food chemistry*, 58(19), 10690–10696. https://doi.org/10.1021/jf102009j
5. Cristiano, G., Murillo-Amador, B., and De Lucia, B. (2016). Propagation Techniques and Agronomic Requirements for the Cultivation of Barbados Aloe (*Aloe vera* (L.) Burm. F.)-A Review. *Frontiers in plant science*, 7, 1410. https://doi.org/10.3389/fpls.2016.01410
6. Hou, J., Li, A., Wang, G., Qin, X., and Liu, Y. (2025). Metabolomics analysis of Astragali Radix in Shanxi Province: Investigating the impact of various cultivation methods and growth years on metabolite profiles. *Food chemistry*, 468, 142492. https://doi.org/10.1016/j.foodchem.2024.142492
7. Dong, P., Wang, L., Qiu, D., Liang, W., Cheng, J., Wang, H., Guo, F., and Chen, Y. (2024). Evaluation of the environmental factors influencing the quality of Astragalus membranaceus var. mongholicus based on HPLC and the Maxent model. *BMC plant biology*, 24(1), 697. https://doi.org/10.1186/s12870-024-05355-3
8. Zhao, S., Li, X., Wang, Y., Xu, R., Li, X., Liu, J., Hou, X., and Liu, H. (2025). Comparison of the Immune Enhancing Activity and Chemical Constituents Between Imitation Wild and Cultivated Astragali Radix. *Molecules (Basel, Switzerland)*, 30(4), 923. https://doi.org/10.3390/molecules30040923
9. Li, J., Bao, B., Xue, X., Zhang, P., and Liu, D. (2024). Quality comparison of decoction pieces of *Astragalus membranaceus* var. mongholicus from different origins and different processing technology. *Chinese Journal of Ethnomedicine and Ethnopharmacy*, 33(19), 48–53. https://doi.org/10.3969/j.issn.1007-8517.2024.19.zgmzmjyyzz2024190010
10. Shao, C., Lin, H., Jin, X., Li, Y., Liu, Y., and Yao, J. (2023). Historical evolution and modern research progress of Astragali Radix processing. *Chinese Traditional and Herbal Drugs*, 54(15), 5057–5074. https://doi.org/10.7501/j.issn.0253-2670.2023.15.030
11. Xu, S., Zhuang, Y., Zhang, Y., et al. (2024). Herbal textual research on Bruceae Fructus in famous classical formulas. *Chinese Journal of Experimental Traditional Medical Formulae*, 30(7), 11–19. https://doi.org/10.13422/j.cnki.syfjx.20230448
12. Zhang, W., Xuan, F., Zhang, J., Xu, Q., Jiang, D., Ren, G., and Liu, C. (2025). Evaluation of different ecological regions for cultivation of best quality Bupleurum: a case study from Shanxi, China. *BMC plant biology*, 25(1), 542. https://doi.org/10.1186/s12870-025-06479-w
13. Sun, J., Li, W., Zhang, Y., Guo, Y., Duan, Z., Tang, Z., and Abozeid, A. (2021). Metabolomics Analysis Reveals Potential Mechanisms in Bupleurum L. (Apiaceae) Induced by Three Levels of Nitrogen Fertilization. *Agronomy*, 11(11), 2291. https://doi.org/10.3390/agronomy11112291
14. Son, T., Chung, I., Lee, S. C., Rico, C., Iwaya-Inoue, M., and Inoue, M. (2009). Effects of cultivation temperature on the growth and saikosaponin production of *Bupleurum falcatum* L. cultivars. *Journal of the Faculty of Agriculture, Kyushu University*, 54. https://doi.org/10.5109/14032
15. Liu, D., Wang, Y., Zhang, X., Wang, Q., Wang, G., and Xia, M. (2019). Research progress of processing in production place and processing methods of Bupleuri Radix. *Chinese Journal of Experimental Traditional Medical Formulae*, 25(19), 204–211. https://doi.org/10.13422/j.cnki.syfjx.20191350
16. Shi, D., Zhang, J., Su, B., Dai, Y., and Sun, L. (2020). Investigation on production place processing technology of Bupleuri Radix. *Chinese Traditional Patent Medicine*, 42(8), 2207–2211. https://doi.org/10.3969/j.issn.1001-1528.2020.08.050
17. Song, J. W., Bhandari, S. R., Shin, Y. K., and Lee, J. G. (2022). The Influence of Red and Blue Light Ratios on Growth Performance, Secondary Metabolites, and Antioxidant Activities of *Centella asiatica* (L.) Urban. *Horticulturae*, 8(7), 601. https://doi.org/10.3390/horticulturae8070601
18. Singh, S. P., Misra, A., Kumar, B., Adhikari, D., Srivastava, S., and Barik, S. K. (2022). Identification of potential cultivation areas for centelloside-specific elite chemotypes of *Centella asiatica* (L.) using ecological niche modeling. *Industrial Crops and Products, 188*(Part A), 115657. https://doi.org/10.1016/j.indcrop.2022.115657
19. Chen, C., Yang, L., Liu, S., Li, Y., Ren, Z., and Zhang, F. (2019). Screening of Centella asiatica drying method based on comprehensive evaluation of quality. *Chinese Traditional and Herbal Drugs*, 50(20), 4911–4919. https://doi.org/10.7501/j.issn.0253-2670.2019.20.011
20. Chauhan, R., Singh, S., Kumar, V., Kumar, A., Kumari, A., Rathore, S., Kumar, R., and Singh, S. (2021). A Comprehensive Review on Biology, Genetic Improvement, Agro and Process Technology of German Chamomile (*Matricaria chamomilla* L.). *Plants (Basel, Switzerland)*, 11(1), 29. https://doi.org/10.3390/plants11010029
21. Yao, S., Tan, X., Huang, D., Li, L., Chen, J., Ming, R., Huang, R., and Yao, C. (2024). Integrated transcriptomics and metabolomics analysis provides insights into aromatic volatiles formation in Cinnamomum cassia bark at different harvesting times. *BMC plant biology*, 24(1), 84. https://doi.org/10.1186/s12870-024-04754-w
22. Bandusekara, B. S., Pushpakumara, D. K. N. G., Bandaranayake, P. C. G., and others. (2025). Intraspecies diversity of the bioactive compounds of wild and cultivated Cinnamomum species in Sri Lanka. *BMC Agriculture*, 1(5). https://doi.org/10.1186/s44399-025-00004-y
23. Wang, Y., Mo, Y. R., Tan, J., Wu, L. X., Pan, Y., and Chen, X. D. (2022). Effects of growing Coptis chinensis Franch in the natural understory vs. under a manmade scaffold on its growth, alkaloid contents, and rhizosphere soil microenvironment. PeerJ, 10, e13676. https://doi.org/10.7717/peerj.13676
24. Huang, H., Liu, X., Huang, L.-Q., Yang, Y.-F., Wu, H.-Z., and He, Z. (2014). Level investigation on Coptidis Rhizoma processing methods and product specifications. *China Journal of Chinese Materia Medica*, 39(16), 3085–3088. https://doi.org/10.4268/cjcmm20141616
25. Chavalittumrong, P., and Jirawattanapong, W. (1992). Variation of active constituents of Curcuma domestica rhizomes at different ages. *The Thai Journal of Pharmaceutical Sciences*, 16(2), Article 5. https://doi.org/10.56808/3027-7922.1271
26. Hossain, M. A., and Ishimine, Y. (2005). Growth, yield and quality of turmeric (*Curcuma longa* L.) cultivated on dark-red soil, gray soil and red soil in Okinawa, Japan. *Plant Production Science*, 8(4), 482–486. https://doi.org/10.1626/pps.8.482
27. Cooray, N., Jansz, E., Ranatunga, J., and Wimalasena, S. (1988). Effect of maturity on some chemical constituents of turmeric (*Curcuma longa* L.). *Journal of the National Science Foundation of Sri Lanka*, 16(1), 39–51. https://doi.org/10.4038/jnsfsr.v16i1.8276
28. Cao, L., Zhao, J., Wang, X., Guo, J., Zeng, J., and Li, Q. (2016). Effect of different processing methods on curcuminoids content in Curcumae Longae Rhizoma and Curcumae Radix from different areas. Chinese Journal of Experimental Traditional Medical Formulae, 22(4), 50–56. https://doi.org/10.13422/j.cnki.syfjx.2016040050
29. Zhang, Y., Wang, H., Liu, W., et al. (2004). Applied fundamental research of Echinacea species. *Journal of Peking University (Health Sciences)*, (1), 90–93. https://doi.org/10.19723/j.issn.1671-167x.2004.01.027
30. Zhang, L., Tian, D., Wan, X., Cao, Q., and Ge, Y. (2025). Effects of different soil moisture contents on the growth, physiological characteristics, and chicory acid content of double petaled *Echinacea purpurea* (L.) Moench. *Journal of Zhejiang University (Agriculture and Life Sciences)*, 51(1), 164–176.
31. Hiyama, H., Ozawa, A., Makino, B., Yoshioka, Y., and Ohsawa, R. (2021). Stability and Reproducibility of Ephedra sinica Ephedrine Alkaloid Content and Terrestrial Stem Dry Weight. *Biological & pharmaceutical bulletin*, 44(11), 1781–1789. https://doi.org/10.1248/bpb.b21-00599
32. Matsumoto, M., Hirayama, M., Ohtomi, N., Ohno, T., Nomura, Y., Iida, O., Sugimura, K., Kawahara, N., Tsuchida, T., and Mikage, M. (2015). Influence of genetic factors on the ephedrine alkaloid composition ratio of Ephedra plants. *Journal of natural medicines*, 69(1), 63–67. https://doi.org/10.1007/s11418-014-0863-7
33. Li, H., Ding, X., Zhang, D., An, Q., Jin, Y., and Zhan, Z. (2022). Herbal textual research on Ephedrae Herba in famous classical formulas. *Chinese Journal of Experimental Traditional Medical Formulae*, 28(10), 102–110. https://doi.org/10.13422/j.cnki.syfjx.20212048
34. Kovalenko, I. M., Klymenko, G. O., Yaroschuk, R. A., Fedorchuk, M. I., and Lykholat, O. A. (2018). Optimization of Ginkgo biloba cultivation technology in open soil conditions. *Regulatory Mechanisms in Biosystems*, 9(4), 535–539. https://doi.org/10.15421/021880
35. Guo, J., Wu, Y., Wang, B., Lu, Y., Cao, F., and Wang, G. (2016). The effects of fertilization on the growth and physiological characteristics of Ginkgo biloba L. *Forests*, 7(12), 293. https://doi.org/10.3390/f7120293
36. Zhan, Z., Zhang, J., Huang, W., and Huang, J. (2025). Transcriptomic strategy provides molecular insights into the growth and ginsenosides accumulation of Panax ginseng. *Phytomedicine : international journal of phytotherapy and phytopharmacology*, 143, 156834. https://doi.org/10.1016/j.phymed.2025.156834
37. Kim, W. Y., Kim, J. M., Han, S. B., Lee, S. K., Kim, N. D., and Park, M. K. (2000). Steaming of ginseng at high temperature enhances biological activity. *Journal of Natural Products*, 63(12), 1702–1704. https://doi.org/10.1021/np990152b
38. Karkanis, A., Cruz-Martins, N., Petropoulos, S., and Ferreira, I. (2016). Phytochemical composition, health effects and crop management of liquorice (*Glycyrrhiza glabra* L.): A medicinal plant. *Food Reviews International*, 34. https://doi.org/10.1080/87559129.2016.1261300
39. Jiang, J., Xiao, S., Yan, S., Xiao, J., and Xu, X. (2020). Glycyrrhizae Radix et Rhizoma Processed by Sulfur Fumigation Damaged the Chemical Profile Accompanied by Immunosuppression and Liver Injury. *BioMed research international*, 2020, 5439853. https://doi.org/10.1155/2020/5439853
40. Zhao, Z., Liang, Z., Chan, K., Lu, G., Lee, E. L., and Chen, H. (2010). A unique issue in the standardization of Chinese materia medica: processing. *Planta Medica*, 76(17), 1975–1986. https://doi.org/10.1055/s-0030-1250522
41. Zhang, X. X., Zuo, J. Q., Wang, Y. T., Duan, H. Y., Yuan, J. H., and Hu, Y. H. (2022). Paeoniflorin in Paeoniaceae: Distribution, influencing factors, and biosynthesis. *Frontiers in plant science*, 13, 980854. https://doi.org/10.3389/fpls.2022.980854
42. Chen, X., Zhang, X. Y., Zhang, R. R., et al. (2009). Effects of Mn, Fe, Zn and Cu on growth and paeoniflorin content of Paeonia lactiflora. *China Journal of Chinese Materia Medica*, 34(8), 961–964.
43. Jin, L., Zhao, W. S., Guo, Q. S., Zhang, W. S., and Ye, Z. L. (2015). Study on chemical components distribution in Paeoniae Radix Alba and its processing methods. *China Journal of Chinese Materia Medica*, 40(10), 1953–1959.
44. Ling, M., He, C., Gao, J., et al. (2015). Effects of aeroponics on the growth, yield and quality of Plantago asiatica. *Guangdong Forestry Science and Technology*, (1), 29–33. https://doi.org/10.3969/j.issn.1006-4427.2015.01.006
45. Li, C., Gong, S., Xu, J., et al. (2018). Research progress on chemical composition and pharmacological effects of Semen Plantaginis and predictive analysis on Q-marker. *Chinese Traditional and Herbal Drugs*, 49(6), 1233–1246. https://doi.org/10.7501/j.issn.0253-2670.2018.06.001
46. Xu, W., Luo, Z., Xie, T., Di, L., Guo, Q., and Shan, J. (2021). Research progress on Platycodon grandiflorus and preliminary analysis of its quality markers. Journal of Nanjing University of Traditional Chinese Medicine, 37(2), 294–302. https://doi.org/10.14148/j.issn.1672-0482.2021.0294
47. Huang, W., Lan, L., Zhou, H., Yuan, J., Shui Miao, Mao, X., Hu, Q., and Ji, S. (2022). Comprehensive profiling of Platycodonis radix in different growing regions using liquid chromatography coupled with mass spectrometry: from metabolome and lipidome aspects. RSC advances, 12(7), 3897–3908. https://doi.org/10.1039/d1ra08285j
48. Sugimura, K., Katsuki, S., Arai, R., Kamiya, H., Kawasaki, T., Iida, O., Kawahara, N., Shrestha, S. S., Dall'Acqua, S., and Watanabe, T. (2024). Relationship between the root processing method and quality of herbal medicine of Rauvolfia serpentina cultivated in Japan. *Fitoterapia*, 178, 106132. https://doi.org/10.1016/j.fitote.2024.106132
49. Gong, Z. (2021). Study on the dynamic change of bioactive components of Ginkgo biloba leaves and its processing and utilization (Master’s thesis, Huazhong Agricultural University). *CNKI*. https://doi.org/10.27158/d.cnki.ghznu.2021.000347
50. Xiong, F., Nie, X., Zhao, X., Yang, L., and Zhou, G. (2019). Effects of different nitrogen fertilizer levels on growth and active compounds of rhubarb from Qinghai plateau. Journal of the science of food and agriculture, 99(6), 2874–2882. https://doi.org/10.1002/jsfa.9500
51. Sugimura, K., Katsuki, S., Arai, R., Kamiya, H., Kawasaki, T., Iida, O., Kawahara, N., Shrestha, S. S., Dall'Acqua, S., and Watanabe, T. (2024). Relationship between the root processing method and quality of herbal medicine of Rauvolfia serpentina cultivated in Japan. Fitoterapia, 178, 106132. https://doi.org/10.1016/j.fitote.2024.106132
52. Zou, Y., Yu, L., Wang, Y., Duan, Y., Cheng, P., and Jin, L. (2025). Research progress on quality evaluation and influencing factors of chemical compositions in Rhei Radix et Rhizoma. *Chinese Traditional and Herbal Drugs*, 56(14), 5225–5236. https://doi.org/10.7501/j.issn.0253-2670.2025.14.025
53. B, P. K., Basak, B. B., Patel, V. J., Senapati, N., Ramani, V. P., Gajbhiye, N. A., and Kalola, A. D. (2024). Enriched soil amendments influenced soil fertility, herbage yield and bioactive principle of medicinal plant (*Cassia angustifolia* Vahl.) grown in two different soils. *Heliyon*, 10(3), e24874. https://doi.org/10.1016/j.heliyon.2024.e24874
54. Subramani, N., Dananjeyan, B., Rethinasamy, V., and Vaikuntavasan, P. (2025). Aflatoxin B1 in senna pods from field to storage in Tamil Nadu, India. *Food additives & contaminants*. *Part B, Surveillance*, 18(1), 21–26. https://doi.org/10.1080/19393210.2024.2405899
55. Nabavi, S. M., Marchese, A., Izadi, M., Curti, V., Daglia, M., and Nabavi, S. F. (2015). Plants belonging to the genus Thymus as antibacterial agents: from farm to pharmacy. *Food chemistry*, 173, 339–347. https://doi.org/10.1016/j.foodchem.2014.10.042
56. Li, Y. (2016). Effect of different cleaning agents and packaging on quality of fresh-cut sage and thyme (Master’s thesis, Hebei University of Engineering). *CNKI*. https://kns.cnki.net/kcms2/article/abstract?v=5ykJdPmCibLfOkI2XGiDKtn8f1oVjaeKjHpbnLXfgCn6iTh60kdSUgSBS2FWUkvSuZUn4OmdX8SlLK3juvfh9l_xSdqR5w2Y12-rIPSHW2VhQsufoJvdEgqRube1CW5iOrtS5YbDfySbxZLJq5Ei7JkwZicrNTk7FyBUqKUvw96g2EoPt_3eev7Rcuk1OnAbuITsTAXIPj4=&uniplatform=NZKPT&language=CH
57. Czabajska, W., Jaruzelski, M., and Ubysz, D. (1976). New methods in the cultivation of Valeriana officinalis. *Planta medica*, 30(1), 9–13. https://doi.org/10.1055/s-0028-1097685
58. Jayasundara, N. D. B., and Arampath, P. (2021). Effect of variety, location & maturity stage at harvesting, on essential oil chemical composition, and weight yield of *Zingiber officinale* roscoe grown in Sri Lanka. *Heliyon*, 7(3), e06560. https://doi.org/10.1016/j.heliyon.2021.e06560
59. An, K., Zhao, D., Wang, Z., Wu, J., Xu, Y., and Xiao, G. (2016). Comparison of different drying methods on Chinese ginger (*Zingiber officinale* Roscoe): Changes in volatiles, chemical profile, antioxidant properties, and microstructure. *Food chemistry*, 197 Pt B, 1292–1300. https://doi.org/10.1016/j.foodchem.2015.11.033
